# The Concept of Advanced Multi-Sensor Monitoring of Human Stress

**DOI:** 10.3390/s21103499

**Published:** 2021-05-17

**Authors:** Erik Vavrinsky, Viera Stopjakova, Martin Kopani, Helena Kosnacova

**Affiliations:** 1 Institute of Electronics and Photonics, Faculty of Electrical Engineering and Information Technology, Slovak University of Technology, Ilkovicova 3, 81219 Bratislava, Slovakia; viera.stopjakova@stuba.sk; 2 Institute of Medical Physics, Biophysics, Informatics and Telemedicine, Faculty of Medicine, Comenius University, Sasinkova 2, 81272 Bratislava, Slovakia; martin.kopani@fmed.uniba.sk; 3 Department of Simulation and Virtual Medical Education, Faculty of Medicine, Comenius University, Sasinkova 4, 81272 Bratislava, Slovakia; 4 Department of Molecular Oncology, Cancer Research Institute, Biomedical Research Center of the Slovak Academy of Sciences, Dúbravská Cesta 9, 84505 Bratislava, Slovakia

**Keywords:** human stress, multi-sensor, telemedicine, electrodermal activity, monitoring, interdigital array of electrodes

## Abstract

Many people live under stressful conditions which has an adverse effect on their health. Human stress, especially long-term one, can lead to a serious illness. Therefore, monitoring of human stress influence can be very useful. We can monitor stress in strictly controlled laboratory conditions, but it is time-consuming and does not capture reactions, on everyday stressors or in natural environment using wearable sensors, but with limited accuracy. Therefore, we began to analyze the current state of promising wearable stress-meters and the latest advances in the record of related physiological variables. Based on these results, we present the concept of an accurate, reliable and easier to use telemedicine device for long-term monitoring of people in a real life. In our concept, we ratify with two synchronized devices, one on the finger and the second on the chest. The results will be obtained from several physiological variables including electrodermal activity, heart rate and respiration, body temperature, blood pressure and others. All these variables will be measured using a coherent multi-sensors device. Our goal is to show possibilities and trends towards the production of new telemedicine equipment and thus, opening the door to a widespread application of human stress-meters.

## 1. Introduction

The environment in which we live greatly affects how we feel and how our organism works. Everything around us affects the quality of our lives and health. Nowadays, people rush and forget about their health, they live unhealthily, they eat poorly and they are exposed to unnecessary stress [1,2]. In addition, the COVID-19 infection is currently greatly affecting our health. Many individuals are isolated in a monotonous environment with fear of infection and without the ability to maintain direct social contacts [3]. All of this can greatly increase physiological stress, which later may have an impact on the overall health situation of the population. It is possible that the COVID-19 pandemic will be followed by an increased incidence of stress-related diseases. Moreover, this isolation and keeping away from other individuals bring also new requirements for diagnosis in the home environment without necessity of visiting a doctor. Thus, reliable remote diagnosis could increase the quality of healthcare and speed up procedures. In the field of telemedicine and the development of holters with advanced sensors, significant progress has been made in recent years, and we are reaching a stage where we can move to a higher level in the provision of healthcare.

Stress is not an unusual response of the human body and has accompanied life since its inception. It can even be useful but when it takes a very long time it worsens an individual’s life and it can lead to illnesses. Short-term stress can arouse and push a person for better performance [4], while frequent and long-term stress may damage the organism [5]. An interesting and explanatory comparison can be a parallel with the computer processor. When we overclock “stress” the processor by increasing the clock frequency and supply voltage, we get increased computing power, but we have shortened the life of the processor in the long-term run, because we have pushed it above the allowable limits. Stress monitoring can be very useful for individuals and for easier communication with a doctor. People could use a stress-meter to find out how much stress they are exposed to and try to reduce its negative effects. This would lead to greater well-being and prevent stress-related illnesses.

Our goal is to consider existing and recent knowledge and transform it into the concept of a new stress-meter, suitable for the use in the home environment. The article itself is divided into several sections according to individual physiological variables. In each section, an up-to-date analysis of the current state and upcoming perspectives are performed. In this article, we deal only with selected physiological variables that are suitable for the use of stress measurement by a small wireless monitoring device. Based on these detailed analyzes, we propose the concept of a new stress-meter at the end of the article.

## 2. Human Stress Phenomena

Stress in some forms affect people every day and the World Health Organization calls stress a “21st Century Health Epidemic” [6]. However, what does the word stress really mean? We can look at it from several angles. In the past, stress was viewed differently and this word is still used inconsistently between disciplines. Until the 16th century, this term was used directly for physical injuries. In the 17th century, stress was associated with sadness, misery, and suffering, while in the 18th and 19th centuries, the word stress was understood as tension, pressure, and effort due to the development of physics. In the 20th century, the view of stress was significantly influenced by wars, fatigue from fighting and nervous shocks suffered by soldiers. At the end of the 19th century, on the basis of significant experiments, the view began to focus on emotions, the homeostasis of the organism and stress started to be thought of as a burden that causes changes in mental health and affects a person’s physiology. At the beginning of the 20th century, the response to short-term stress after the secretion of adrenaline was concretized by Walter Cannon, who described prepares the organism for a rapid “fight or flight” response, and who made significant discoveries with respect to internal balance—homeostasis. On the other hand, Hans Selye known as the “father of stress”, focused on the chronic, long-term stress. He pointed to the role of the brain and the adrenal cortex in response to stress and identified several hormones that regulate the stress response. Unlike previous years, he emphasized the importance of psychological and social factors in inducing a stress response. He gave the first definition of stress, where it is referred to the mutual action of forces that take place in any part of the body, physical or mental and which represent a psychophysiological response of an individual mediated primarily by the autonomic nervous system (ANS) and the endocrine system [6,7,8,9]. Selye also described the “General Adaptation Syndrome” [7,9], which deals with changes in the body after exposure to stress, arguing that each stressor factor stimulates the same response in the body. Today, stress can be described as “any effect of a change in the environment on a living being that results in disruption of the homeostasis of that living being” [9].

Nowadays, stress is categorized mainly as acute (short-term) and chronic (long-term) [10,11,12]. The human body responds differently to various durations of stress stimuli Acute stress can be for example a job interview, a fine for speed and more. Certainly, acute stress is unpleasant, but the reaction can be positively influenced by soothing breathing or rapid physical activity [13,14,15,16,17,18]. The problem arises when the stress is too intensive, the stressors accumulate and one cannot get rid of them. Persistent chronic stress may have a milder course, but the body is prepared for a stress response long time. Stress hormones are released and the body does not recover as fast as when it is at rest. This can lead to more severe physiological manifestations than those of acute stress. People might feel headaches, insomnia, fatigue, inattention, digestive problems, and memory impairment can occur [19,20,21,22,23,24,25,26]. Each individual respond to stress differently. Effective stress management involves the identification and management of both acute and chronic stress. And it is precisely to be aware of the stress response and subsequent compensation that it is possible to monitor a person’s physiological condition and change life for the better and predict disease [27]. While acute stress can stimulate a person to perform better, in chronic stress performance decreases rapidly. This also applies to stress in the workplace. Not only a person has physiological manifestations of stress, but also stress is reflected in the results of work done, loss of productivity, burnout, dissatisfaction with work and others [28,29,30,31,32]. Solving the issue of stress is very important from a human and economic point of view. According to The American Institute of Stress, work stress is a major source of stress for adult Americans and is on the rise. They reported that approximately 33% of people experience extreme stress. Stress is responsible for 80% of accidents and 120,000 deaths per year in the workplace. The Global Organization for Stress reported 75% of Americans and 91% of Australians exposed to stress. At work, 80% of American and 86% of Chinese workers experience stress. They are also reported 450,000 workers in Britain with stress-related illnesses. About half of people exposed to stress are affected by post-traumatic stress disorder (PTSD) and acute stress disorder. The downside is that it tends to get worse and no one is completely resistant to stress. An increasing number of people exposed to stress and the contraindications associated with stress show us that there is a need for equipment and methodology that would help detect stress and fluctuations in mental health and help reduce them [19,33,34,35,36,37,38,39,40,41]. The stress is affecting also the economy, and U.S. employers are spending health care and working days at $300 billion a year. In Britain, people miss out on 13.7 million working days a year because of the stress, and it costs them about $37 billion, and in Australia, stress is responsible for the loss of $14.2 billion. Stress in everyday life and in the workplace is related to anxiety and depression. Statistically, more than 300 million people suffer from depression and, along with anxiety, are the most common mental disorders. The annual global cost is estimated at $2.5 trillion and is very likely to increase in the coming years [35,36,37,39,40,41,42].

Stress from the perspective of medicine shows how stressors stimulate the human body to defend itself. The response to stress affects the whole biological system of the organism and physiological processes. This is manifested by various symptoms, often deleterious individual problems such as headaches, gastrointestinal disorders, anxiety, hypertension, coronary heart disease and depression [43]. It should be mentioned that the response on stress begins in the same way. During the stress response, the stress hormones adrenaline and noradrenaline, which are released by the sympathetic nervous system, and cortisol that is produced after activation of the hypothalamic-pituitary-adrenal axis, are released [44]. Cells in the body express receptors for stress hormones, so they are easily provided with information about the stress stimulus. Other factors such as adrenocorticotropin (ACTH), oxytocin and vasopressin, cytokines (interleukin-6 and interleukin-1β) also play a role in stress. The length and magnitude of action of these factors depend on the stressor type. Overall, the response to stress is still biologically consistent and these physiological symptoms are suitable for measurement [16,23,24,45,46,47,48]. Autonomic nervous system (ANS) plays main role in the response to stress, which cannot be controlled by our own will. It consists of sympathetic and parasympathetic systems; whose balance regulates the physiological degrees of “arousal” in the response to signals from the environment. The parasympathetic regimen regulates the maintenance of energy and the renewal of the organism; sympathetic one stimulates increased heart rhythm, blood pressure, dilated pupils, sweating and other physiological manifestations caused by the secretion of adrenaline and norepinephrine. These manifestations are visible even under stress [49], so we focused on the possibility of recording them as well as on the subsequent interpretation of a person’s condition [27,50,51,52,53,54]. The primary response to stress triggers a sympathetic nervous response that regulates the adaptation to the external environment. The hypothalamus secretes hormones that stimulate the pituitary gland and thus begin to regulate the stress response. The pituitary gland secretes adrenocorticotropic hormone (ACTH) into the blood. This hormone helps to balance the intensely regulated stress response. The amygdala, in turn, regulates emotional processes. Activities in the prefrontal cortex are temporarily suppressed (planning, attention, problem solving). Adrenaline and norepinephrine and cortisol are excreted. Serotonin, which regulates mood, especially depression and anxiety, is also secreted. The hypothalamic-pituitary-adrenal (HPA) axis enables the communication of three endocrine glands (hypothalamus, pituitary, adrenal gland), which ensure the stress response, but also digestion, mood, autoimmune system, energy, sexual cycles [33]. Inflammatory markers eluted approximately 90–120 min after the onset of acute stress, specifically IL-6, IL-1β and TNF-α. Acute stress induced an increase in the pro-inflammatory transcription factor NF-κB with the highest amount after 10 min. Chronic stress shows less inflammation, and available studies show elevated levels of TNF-α and CRP over an observation period of 3 years. Inflammatory cytokines and CRPs may play a role in increased amounts of secreted cortisol [55].

Also interesting is the connection between stress, CNS, ANS and voice output, which consists of three stages—breathing, phonation and resonance. Thus, ANS is responsible not only for the stress response but also for the creation of voice and speech. Even a shaking voice is our body’s response to stress. The voice can be a very sensitive indicator of a person’s emotions, attitudes, mental experience, depression, anxiety, tremor or physical fatigue. Voice quality can be the result of tension throughout the body, which also manifests itself locally as a specific tension in the external and internal laryngeal muscles [56]. It’s all about the vocal cords, which in this case affects the ANS. There are currently several studies that attempt to identify stress patterns of voice using neural networks and machine learning [57,58,59]. Some even combine speech signals with electrodermal activity [60] or use wearable devices with multi-sensors combining audio and physiological sensors together with deep neural learning networks to monitoring an individual’s well-being in a naturalistic environment [42,61,62].

In order for stress to begin, an individual has to be first be exposed to a certain stressor, which initiates a stress response. Anything that forces the body to release stress hormones can be a stressor and cause stress. Stressors can be divided into psychological and physiological. Physiological effects on the body include, for example, very high/low temperatures, injuries, chronic diseases, infection or pain. Psychological (mental) stressors can be attributed to life situations, negative social communications, conflicts, failure to satisfy internal drives and others [17,19,63,64]. The division into physical and psychological stressors is also based on the scientific function of the organism, because these various stressors activate every other part of the brain, and the trace they leave can be used back to concretize the stressor [17]. On the other hand, stressors can also be divided into absolute stressors, which would be evaluated by everyone who is exposed to them as stress (objective stress factors such as natural disasters) and relative stressors, where we can include effects that only part of the population would declare as the stress initiators (subjective stress factors such as time pressure, tax payment, school exam) [11,16,65,66,67,68]. However, all stressors activate the same biological response of the body [44]. To determine the stress to which a person is exposed, the “Social Readjustment Rating Scale” (SRRS) was released in 1967 [69], but it was not ideal because it offered a very subjective view.

The stress monitoring techniques used today are based on several variables as self-assessment, measurement of behavior and cognitive functions, and finally on physiological manifestations. In our study, we focus mainly on physiological manifestations, because they are an objective manifestation of the organism [28,32,49,70,71].

Stress affect all body system, including muscles, respiratory, cardiovascular, endocrine, gastrointestinal, nervous, reproductive systems and speech [23]. Therefore, it is possible to measure these physiological signals of the body, and after combining all the outputs together, we can get a correctly determined result of the stress level. In addition, monitoring people in high-risk occupations where they are exposed to stress on a daily basis or nowadays people quarantined and without direct social contact due to COVID-19, can help preserve their health and prevent possible illness. In this way, chronic stress can be prevented, individuals can protect themselves more effectively and compensate for the time during which they feel stress. The measured physiological values can also serve experts in the observation and analysis of psychophysiological changes in the body and on the basis of the obtained data, diagnostic and therapeutic procedures can be optimized [72,73,74].

## 3. Physiological Variables in Stress Measurement

### 3.1. Electrodermal Activity

Electrodermal activity (EDA) very well reflects the activity of sympathetic ANS [3] and is insensitive to the parasympathetic system. It is purely a measure of sympathetic activity. EDA is a technically simple and very popular method for determining the psychophysiological response of the body known since the 19th century [75] and it is widely used in clinical research and in various cases such as understanding emotional behavior, the effects of dysregulation, alcoholism, anxiety, autism, ADHD, depression, reactions to various types of addictions, epilepsy, menopause, pain, phobias, psychiatric counseling, schizophrenia, sleep monitoring and disorders, fatigue, exercise recovery and sociopathy. This method is very sensitive, and it can detect even small changes from the external environment. Moreover, it fits perfectly for stress measurement [49,76,77,78]. Nowadays, EDA is used mostly to measure acute stress, however first devices for EDA recording of chronic stress appear [79].

It has been found that skin becomes a better conductor of electricity for a while after exposure to external stimuli [2,5,23,80,81,82,83,84,85,86,87,88]. EDA methodology is based on innervation the sweat glands in the skin by sympathetic nerves, which control the synthesis of sweat and its excretion [81,89]. The activity of the sympathetic nerves is mostly sensed by measuring of the electrical conductivity between two electrodes placed on skin surface of bottom part of the hands, especially fingers or feet, where most nerves are located [90,91,92,93]. Such an EDA method is called exosomatic. It is obviously preferred method and we will also use it in proposed concept. Measurements need a direct (DC), commonly used, or an alternating current (AC) source. DC can provide full EDA signal [94], but electrode polarization can be a problem [81]. EDA can be measured also passive—endosomatically, where potential activity is obtained [81,88,93,95,96,97].

**Figure 1:**
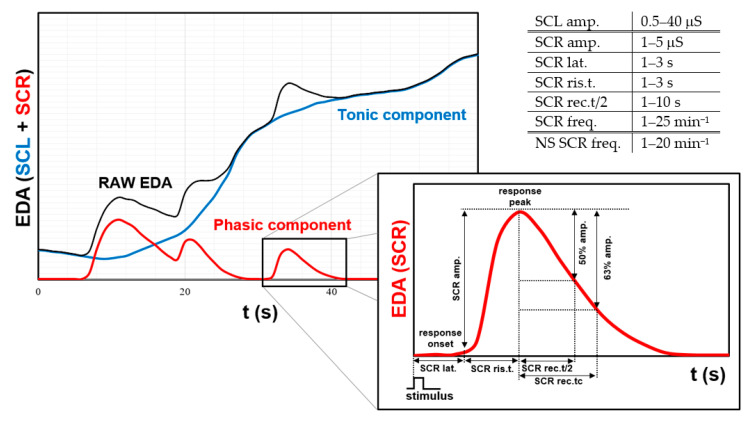
Typical EDA response.

The EDA signal consists of a tonic response (skin conductivity level, SCL) as well as a rapid phasic changes (skin conductivity reaction, SCR) (Figure 1) and artifacts that also form a permanent part of the EDA data [98]. SCL represents the basal activity of the sympathetic system [99,100]. Absolute value of SCL is individual. It should be noted that a hand-held lumberjack will have a significantly different absolute value of skin conductivity SCL than a small child. Therefore, we are only interested in variations, changes and the shape of the curve of this particular person’s signal, because it is already related to long-term stress. Even in situations where we do not have information about stimuli and we measure stress for a long time, it is appropriate to use the SCL parameter. Typical SCL is between 0.5 and 40 μS. The phasic response of SCR reflects changes to a discrete unexpected stimulus and events [83,101]. For example, the stimulus may be a cognitive and emotional response that causes activation of brain regions such as the amygdala, hippocampus, basal ganglia, and prefrontal cortex. Typical SCR occurs between 1 and 3 s after stimuli (SCR lat. in Figure 1) (rarely 5 s), the rising time (SCR ris.t) is in range 0.5 to 5 s (typical 1 to 3 s) and the SCR half recovery time (SCR rec.t/2) is between 1 and 10 s. SCR amplitude (SRC amp.) is normally in range 1 and 5 μS but under high aversive, threatening or fearful stimuli can reach up to 8 μS. The overall duration of one SCR is between 10 and 20 s, followed by a return to the tonic level. Typical response frequency is 1–10/min, if we are relaxed, and can reach 25/min under high arousal. Spectral content of EDA is mostly confined to 0.05–0.15 Hz. During intensive exercise about 0.37 Hz. Non-specific SCR (NS-SCR) response could be also used. Its frequency varies according to the situation from 1–3/min at rest to more than 20/min in exciting situations and therefore this component could also be used as an indicator of stress [83,88,92,93,102,103,104]. Phasic EDA output is used mainly for short-term measurements with precisely defined stimuli, which is very suitable for laboratory experiments. But we think that by adding multi-sensors and a comprehensive analysis of physiological variables, it will be possible to use this component even in an area where long-term monitoring is necessary.

Another interesting idea is the use of EDA-recorded emotional patterns to detect emotions and find abnormalities through machine learning and neural networks. Today, this expanding industry can add robustness to the classification of human emotions without additional physiological variables. Emotion recognition can help older or disabled people become more involved in life, e.g., Active and Assisted Living (AAL) and Driver Assistance Systems [105].

#### 3.1.1. Optimization of EDA Measurements for Wearable Devices

Electrode optimization, whether by appropriate dimensional adjustment or by the choice of materials used, is an important process for improving the output signal. Properly optimized electrodes can also minimize artefacts that interfere recorded signal. In order to obtain a correct and sufficiently strong signal, we need suitable electrodes which do not polarize during the transition of the current and which show a minimum bias potential (between pairs of electrodes) [98]. The quality of the skin-electrode contact is essential but wearing comfort is important too. Kim et al. [80] produced a soft wearable sleep monitoring system with flexible electrodes produced by nanomaterial printing which obtained graphene layers. These layers were transferred into a silicone flexible and breathable tape. This flexible approach also allows suppressing the movement artefacts during sleep. Another study [106], focused on the design of electronic textile Ag/AgCl electrodes on cotton, nylon and polyester fabrics brings interesting optimization. Textile electrodes do not yet achieve the signal strength of ordinary electrodes but they bring an advantage in the form of air permeability, comfort and invisible EDA monitoring systems (University of British Columbia, Vancouver, Canada). Also interesting is the idea with the new GEDIS system (Graz Electro Dermal Impedance measurement System), which measures and analyses the electrical impedance allocated on 2.5 × 3.5 cm matrix. The matrix consist of 48 spring-mounted gold-plated beryllium copper electrodes and manages to measure impedance of the skin at different parts of the human body even continuously for several hours [107].

**Figure 2:**
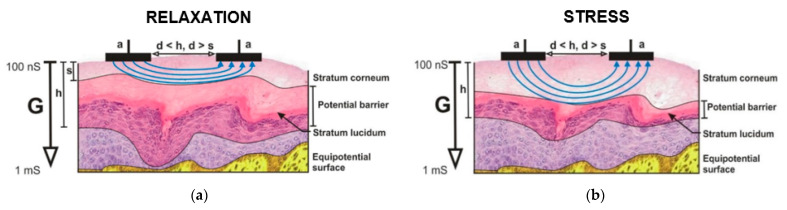
The dominant vector intensity lines of the electric field inside of human skin by using microelectrodes: (**a**) in relaxation, (**b**) under stress stimulus.

Nowadays, all wearable systems measure skin conductivity at a macroscopic level using relatively large electrodes. That means the electric field is closed perpendicular to the surface of the skin. The signal passes from the first electrode across all skin layers into vessels, then through whole length of vessel back to the next skin structures and the second electrode. The imperfection of this system is the fact that it also records all stress independent fluctuating parameters that actually occur in the skin and blood vessels. The resulting EDA signal is thus disturbed by adverse effects [71,108]. Therefore, we began to investigate whether these fluctuations could be minimized. It was generally assumed that the increase in skin conductivity during the stress response was caused only by sweating. Later the existence of a potential barrier near the stratum corneum [109] (Figure 2) and stratum lucidum [110,111] was found. Dynamic electrical properties of the potential barrier reflect the fact that it is responsible for trans-epidermal transports—substance exchange, water transport, thermo-regulation and immunology [112]. The thickness of the barrier and its properties can vary due to the activity of the sympathetic nervous system and stress influence [113,114,115].

Therefore, we decided to develop a device that would measure EDA only in these upper layers of skin. For this purpose, interdigital array (IDA) microelectrodes have been used. We know from the simulations [110,111] that when using symmetrical IDA microelectrodes, the depth of penetration of the electric field into the skin is approximately equal to the distance between electrodes. If the distance between electrodes is less than the thickness of the electrically active skin layers, the lines of the electric field are parallel to the laminar structures of the epidermal skin. From inner layers of skin, the electric field intensity lines are embossed to the surface (to the area with a lower conductivity) by the influence of the potential barrier which is generated by electrical double-layer around stratum lucidum (Figure 2a). Under a stress stimulus the potential barrier narrows down and the electric field can reach inner layers of human skin with higher conductivity, and the total conductivity increases (Figure 2b) [116]. In our research IDA microelectrodes with utilized dimensions between 100 μm/100 μm and 200 μm/200 μm (finger/gap) were used for laboratory tests.

**Figure 3:**
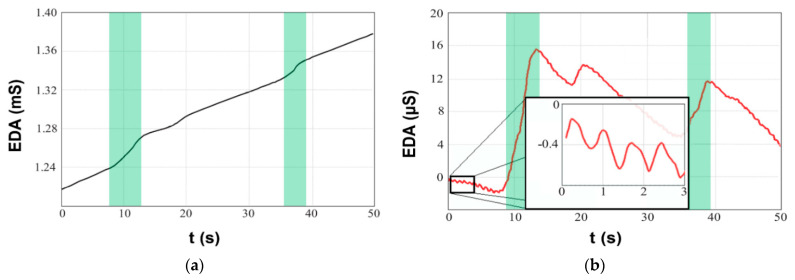
EDA obtained using IDA microelectrodes: (**a**) tonic component, (**b**) extracted phasic component with small variations due to blood pulse wave.

We have determined the appropriate amplitude, shape and frequency of the input signal for selected configuration of EDA microelectrodes. The optimum amplitude of the input signal should be in the range from 1.5 V to 3 V. The frequency of the input signal is not so critical, nevertheless a value in the order of ones of kHz has been proved as the optimum. During the measurements, we also mathematical separated the tonic response (Figure 3a) and phasic response (Figure 3b) of the EDA signal. However, as this terminology had not yet been very well established at the time, parameters G and ΔG can be found in our older sources [116]. There was one interesting observation found, we sometimes managed to capture the pulsation of blood in the arteries on variations of the conductivity itself. We assume that this was caused by mechanical compression of the skin layers and by a change in the quality of the electrode-to-skin contact.

**Figure 4:**
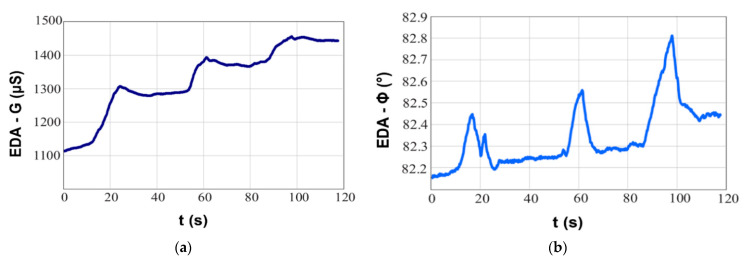
EDA—complex impedance measured using a 10 kHz harmonic input signal: (**a**) conductivity (**b**) phase.

In contrast to conventional measurements, we have also enriched some experiments to measure not only conductivity, but also complex impedance and its dependence on the source frequency. We observed that the absolute value of the impedance decreases with increasing frequency of the input signal. Therefore, some compromise between these two parameters is necessary. An interesting outcome was observed, EDA can also be sensed accurate by the skin admittance phase, as this parameter significantly reflects the human skin conductivity changes. A harmonic signal with a frequency around 10 kHz has been found as the most proper input signal (Figure 4). Unfortunately, there are only few works available, where the influence of frequency and complex impedance of EDA has been investigated. We found only one study focused on the signal frequency variations in the range 0 to 1 kHz using a sensor glove with textile electrodes placed on fingers [96].

**Figure 5:**
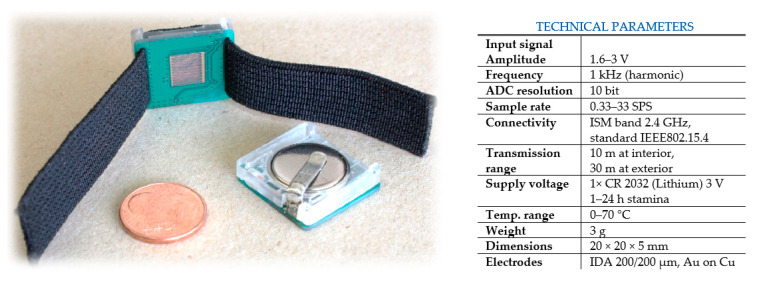
EDA-ring: design and technical parameters.

After a period of laboratory testing and several design steps, we finally built a simple wearable EDA system implemented in the form of a ring with optimized IDA electrodes placed on the bottom part of finger, where the EDA signal was transmitted directly by bluetooth (Figure 5). All experience gained through this research has been transferred into our current concept of an advanced stress meter.

#### 3.1.2. Electrode Motion Artifacts

Detection of artifacts and noise in EDA signals is required to obtain a quality signal for analysis. These artifacts need to be minimized and further filtered, smoothed or manually deleted. Getting rid of artifacts in laboratory conditions is relatively easy. The tested person is monitored and undesired movement can be easily restricted. With the recent trend of wearable devices and real-time measurements in the home environment, the demands for accurate measurement, analysis and automation are increasing [98].

EDA analysis is especial sensitive to motion artifacts that might degrade the measured signal. This is more visible when using dry electrodes, which we also use in our concept. On the other hand, dry electrodes are more practice from long-term measurements point of view and additionally, they do not degrade over time [81]. Several publications deal with the minimization of motion artifacts with the help of signal filtering and software applications [90,91,117,118,119]. For example, the study of Posada-Quintero [81] describes corrections of artifacts using a stationary wavelet transform and filter transformation of the curve. In the research works Wavelet-based [120] and extended Kalman filter [90] motion artifact removal are mentioned. In the already mentioned article by Kim et al. [80] soft adaptive electrodes were used to solve motion artifacts problem.

However, there are also attempts to suppress motion artifacts in EDA by append additional component and accelerometers come into consideration first. Today’s availability and implementation is at a very high level. Although this research, which examined 8 different types of machine learning in the EDA and accelerometer signal database, suggests only a very low (0.4%) contribution to the resulting EDA signal quality [119]. The disadvantage may be that the employed device Affectiva Q Sensor (Affectiva, Inc. Waltham, MA, USA) uses two relatively large circular electrodes located at the bottom of the wrist. Wrist exhibits very irregular movement with any activity of fingers, and especially the palm. Unwanted blows with the palm of the hand to the device itself are also very undesirable. In the past, we performed a parallel set of EDA measurements on the wrist top and bottom [116]. Although we used microelectrodes with the total area of only about 1.5 cm^2^, the measurement on the underside of the wrist was good only if hand movement was completely disabled. Thus, we would certainly test the use of accelerometers for other hardware and electrode placement configurations. These would definitely be used at least to determine the time of increased motion artifacts, as some researchers did with a simple 2-axis accelerometer [121]. Very good results of minimizing motion artifacts were achieved with differential approach using two sensors in parallel. One was placed on the left hand, the other identical on the right and the resulting EDA was derived [122]. It is also a good idea to compare the palms and distal forearm sites and select the electrode material by comparing the conductive fabrics with Ag/AgCl electrodes [77]. The addition of a third electrode to endosomatic (potential) EDA measurement [91] or the use of a piezoelectric sensor to suppress motion artifacts also seems to be very promising. Although it has been implemented in PPG sensors [123]. The ability to measure the time between transmitting and receiving an optical radiation would also be a challenge, since the reflection occurs at the interface of the walls of the bloodstream. Thus, we would be able to suppress even the biggest error of EDA measurement, and that is the fluctuation of the pressure between the electrode and the skin.

### 3.2. Heart Rate, Heart Rate Variability and Electrocardiography

Heart rate (HR) measurement is a common method to determine the physical activity and condition of the body. The HR reflects the overall activity of the ANS and this activity changes provides a suitable indicator of the human state and mood. Under stress, the heart rate is increased [78,124]. Heart rate variability (HRV) is derived from HR. Actually, it is a measure of the variation in time between each heartbeat [125,126]. By contrast to EDA, HRV reflects the function of both the sympathetic system and the parasympathetic systems. The usage of HRV analysis is widespread, not only for medical use, but also for measuring emotions, thoughts, behavior or feelings. In acute stress, the HRV value is low, on the contrary, during rest is high. Low HRV value is also associated with the development of cardiovascular disease and an increased risk of death and it is also linked to increased arousal, illness anxiety and emotional disorder. High HRV indicates higher action readiness, and higher resistance to stress and stress recovery and is associated with higher self-control skills, better stress management skills and greater social skills [127,128,129,130,131]. Acute and chronic stress have negative impact on the heart and thus this fact is manifested on HR and HRV. Acute stress increase HR and sympathetic activity decrease HRV. Chronic stress also increases HR [132], while HRV is low [131].

It is generally accepted that the activities of the ANS, are reflected in the low-frequency (LF: 0.04–0.15 Hz) and high-frequency (HF: 0.15–0.4 Hz) bands of HRV. On the other hand, not without some controversy, the ratio of the powers in those frequency bands so called LF-HF ratio (LF/HF), has been used to quantify the degree of sympathovagal balance [133]. The most significant recent work may be considered R. Baevsky, who proposed detailed the methodology for HRV analysis and special determination of health and psychological condition for many years [134]. The stress level is determined by the Baevsky stress index and it is widely used today [135,136]. An interesting view based on more precise levels of LF and HF, not only their ratio is also mentioned by the authors in [130].

We have also investigated whether the stress manifests itself in any anomalies on the ECG curve. Demonstrable change in the incidence of atrial fibrillation has been found only at very high levels of acute stress and anxiety [137,138]. Certain changes are observable also on the T and P waves [139]. We also have some weakly proven references about changes in the P wave in choleric patients, and the study of broken heart syndrome [140] is also interesting. In general, we do not consider ECG curve abnormalities to be significant for stress-meter development purposes. However, ECG measured with a chest holter is a great source for HRV analysis, and in general, the ECG holter is still the most important device in telemedicine. HR itself can be obtained by a very wide range of measurement methods. Most commonly, it can be obtained from the biopotentials of the heart and using photo and piezoelectric plethysmography. Our experience include also obtaining HR from SCG seismocardiography [141] or from minor variations of the EDA itself [116].

### 3.3. Circulatory Shock—Temperature and Skin Reflectance

Another important and easily measurable parameter manifesting stress is the body temperature. Temperature in stress response is controlled by ANS. There is always a change in temperature during stress, however it does not react as fast as EDA. The body has a high thermal capacity and thermoregulatory processes require some time, but the thermal sense represents one of the best characters of state of physical relaxation. Temperature changes are inflicted due to increased amount of blood flowing in bloodstream by vasodilatation that causes hyperthermia or even by vasoconstriction and related hypothermia [142,143]. The studies discovered both responses to stress stimuli [144]. The activity of sympathetic nerves mostly increase temperature [145,146] due to sympathetic control of noradrenaline secretion [146,147]. Studies describes decreases temperature in the fingers after stressor stimuli [148] or refer decrease in hypothalamic temperature and ear skin temperature [146]. Acute stress showed short-term drop in skin temperature, because it triggers initial peripheral vasoconstriction [149]. Stress have impact to the peripheral and body core temperature. While peripheral distal skin temperature has tendency of decreasing, core temperature increases [142,150]. During chronic stress, even the fever can be developed [151]. To summarize, with increasing stress, the core temperature of the human body or partially also face (which may be also related to emotion expressions) increases [152], while the temperature of the periphery of the human body (fingers) decreases significantly (a decrease of 13.5 °C was reported) [142,149,153]. The core temperature responds more to the overall physical and mental activity, while the peripheral temperature reflects the current stimuli [154].

We would venture to compare this behavior of periphery vasoconstriction to the so-called “circulatory shock”. Circulatory shock is expression of the circulatory failure and inadequate oxygen level [155]. It does not occur on such a large scale as in the case of blood loss or hypothermia, but the wisdom of our elders about stress and fear has a tiny scientific basis: “Blood would not cut in it” or “He turned pale/green with fear”. So indeed, even during slightest stress or danger, small extent of circulatory shock occurs. Therefore, we recommend equip the portable stress-meter ideally with at least two temperature sensors—one for the body core temperature, the other for the peripherals and possibly another measuring the outside temperature. Partly, they did so in [156], where they introduced a quantity that depends on the ratio of the temperature of the fingers and the face. We would rather replace the face temperature with a more suitable core body temperature measured, for example, on the chest [151].

The technical implementation of temperature measurement is rather simple. We can use contact sensors such as thermocouples, metal sensors, diodes, double-transistor measurement, etc. or non-contact optical measurement. When selecting/manufacturing a sensor, it is important to select the appropriate temperature range so that the sensors are stable and have the highest possible sensitivity in the human body temperature range. Perhaps more important than their absolute accuracy will be ability to respond to small temperature variations. We might also pay attention to the asymmetric ratio temperature sensors [157].

**Figure 6:**
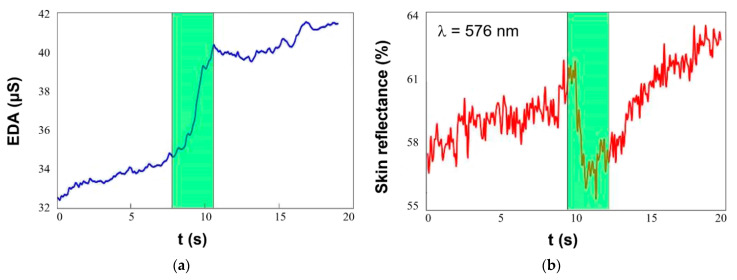
Simultaneous electro-optical measurement of stress: (**a**) EDA, (**b**) skin reflectance.

In the meantime, one can provide experiments with measuring the reflection and spectral characteristics of the skin. The dermis is heavily permeated with blood vessels that contain hemoglobin, which has a unique light absorption spectrum with characteristic absorption bands in the 540–576 nm wave length range. Using optical (light reflectance) measurements, we were able to monitor the quantity of hemoglobin in top layers of the human skin. Coherence between the EDA and the reflection of skin in the bottom area of fingers and wrist (Figure 6) were investigated. The shape of the reflectance curve roughly copies the shape of the EDA resistivity. This actually confirms the presented theory of so-called “circulatory shock” due to stress. Due to short-term stress, blood from the periphery moves to more important internal organs, which causes a drop-in temperature and reflection in these places.

To measure the skin reflectance, one has to focus on the wavelengths of hemoglobin absorption spectrum. It would be interesting to combine a hemoglobin sensor with a temperature optical sensor with actual reflective pulse-oximeters (PPG), which are present in almost every smart watch or bracelet today. It may lead to a unique and robust optically based sensor for the measurement of temperature variation, hemoglobin presence, heart rate and blood oxygen saturation spO_2_. The spO_2_ is measured on basis of hemoglobin bound to the oxygen and on different wavelengths of light-red (660 nm—deoxygenated blood) and near-infrared (890 nm—oxygenated blood) [158,159,160,161]. In rare cases e.g., (people suffering from chronic anxiety) a stressful situation can cause respiratory complications and subsequent depletion of spO_2_. Oxygen depletion in elite sports can also act as a stressful factor. However, both of these areas are not our primary focus, so we do not consider spO_2_ sensing necessary since having fundamental information from respiratory sensing [162]. To summarize, one can conclude that stress is a factor, which have an impact on heart, circulation and oxygen transfer and could show symptoms of circulatory shock [163,164,165].

### 3.4. Respiration

Stress sympathetic activation also affects the respiratory rate (RR). Respiration frequency increase and is less stable during the stress response. We can use this knowledge to increase reliability of the HRV stress marker [166]. Also high correlation between emotions, especially anxiety levels and respiratory rate was observed [167]. In this research, the authors divided the experiment into two phases: mental stress and physical exertion while analyzing: minute ventilation V_E_, tidal volume V_T_, respiratory rate RR, O_2_ consumption and CO_2_ production were analyzed. Unpleasant emotions caused by mental stress altered the breathing pattern. V_E_ increased in all subjects during mental and physical exertion, however for subjects with high anxiety, RR increased more than V_T_. Similar results were observed also during quiet breathing [168]. It is also known that a person who concentrates on a certain role breathes shallower and V_T_ is decreased. In [169], the authors investigated mental stress using multi-variate time-frequency analysis of cardiorespiratory.

A very good overall review about impact of stressors on the contribution of RR, V_T_ and V_E_ was written by Michael J. Tipton et al. [170]. He claims that ventilation is increase in response to stress in humans and he also reported it as an increase in V_E_. Identical V_E_ can be achieved by a wide variety of changes in the depth V_T_ and number of breaths RR. It seems that more intense stress leads to an increased V_E_, which increases more by RR than V_T_, disregarding whether this was the case in the lower stress case. Stress-induced ventilation depends on the nature of the stress, so that metabolic-chemical/chemical stimuli usually lead to an increase in both RR and V_T_; and non-metabolic/non-chemical intense stimuli (thermal, nociceptive, psychological) to increase RR. Exposure to environmental stressors is often associated with simultaneous exercise, which may be related to the response of V_E_ to environmental stress, which is suppressed by physiological manifestations of exercise. In addition, exercise also gradually increases body temperature, can increase heart rate and respiratory rate. People can influence the generation of respiratory patterns if they play wind instruments, talk or swallow. As shown, dependence of respiration on stress is rather complex, and one must examine also other physiology, so again use a multi-sensor system. We have to consider that the respiration itself is influenced by the posture, so the use of IMU sensors is necessary.

As for the measurement of respiration itself, the use of chest straps is rather common. For our purpose of holter measurement, however, we recommend using impedance respiration measurement. From the previous experiments, we have experienced that the results in terms of RR, V_T_ and V_E_ are not only comparable, but in some situations, such as increased physical activity is their function more reliable [141].

### 3.5. Blood Pressure

The impact of stress on blood pressure (BP) is involved with a response of the sympathetic nervous system by releasing catecholamines. These hormones temporarily increase blood pressure by making the heart to beat faster and narrowing blood vessels. The stress-related increase in blood pressure can be dramatic and are present in short-term peaks. Such sympathetic responses to acute stress are well documented, but the process by which stress contributes to a sustained increase in BP over time is not well known. It may be repeated activation of this system, inability to return to a resting level after stressful events, failure to become accustomed to repeated stressors, or a combination responsible for the development of hypertension [171,172]. Stress can cause hypertension by repeatedly raising blood pressure as well as stimulating the nervous system to produce large amounts of vasoconstrictive hormones that increase blood pressure [173]. The application of the classical Riva-Rocci method for measuring the BP and the use of a pressure cuff in the concept of an advanced stress-meter is out of the question. Fortunately, PPG has been widely used in recent years to determine cuff-less blood pressure. This principle calculates systolic and diastolic BP from the PPG curve using dedicated algorithms [174]. Currently, more and more devices are beginning to appear that measure the approximate blood pressure by a phase shift between the ECG and the PPG curve [175,176,177,178]. We consider this approach to be suitable for use in the proposed advanced stress-meter.

## 4. Advanced Wearable Stress-Meters

Current human stress monitoring devices are usually very inaccurate or rely on a strictly controlled laboratory environment. The concept proposed in this paper is based on our long-term experience in this scientific field in which we have been operating for the last 10 years [179]. In this work, we focused on a multi-sensor device, because one separate sensor is not able to determine whether a person is really exposed to stress. Simply put, the electrical conductivity of EDA actually only responds to brain activity. Using memory is less energy-intensive than inventing new things. As an example, a lie detector can be used. It works on the principle that when person telling the truth, the brain is not so burdened (EDA drops) as when a person lies, invents and the brain is more congested (EDA increases). The EDA does not distinguish whether the increase in conductivity actually occurs due to real stress or only increased physical or mental activity. A single sensor does not know if the change in finger temperature is caused by stress or the outside temperature. It does not know if posture affects respiration or HRV. Therefore, a reliable and accurate stress monitor that includes not only long-term stress but also acute short-term stress must be multi-sensor. The result must be supplemented by other physiological variables in order to obtain the most comprehensive view of the overall situation of a subject under test. Our aim is to create a multi-sensor device for sensing several physiological variables. An advanced stress meter must be something like a portable lie detector, which also captures blood pressure, pulse, respiration in addition to skin conductivity.

### 4.1. Multi-Sensor Monitors Overview

A large number of scientists have been interested in the idea of using the coherence of several sensors to determine stress in their laboratories [148,180,181,182]. For example it is worth to mention the classifier of negative emotion induced by a visual stimulation evaluated from EDA, ECG and skin temperature [183], multimodal emotion evaluation from combination of EDA, ECG and EMG [184], driver anxiety detection using EDA, PPG, EEG and pupil information [185], identification of cognitive tasks by machine learning from EDA and HRV [186], evaluating of mental workload during web browsing from EDA, PPG and EEG [156,187]. Nowadays, the trend is the use of virtual reality [145,188].

In the current scene of wearable devices usable in the diagnosis of human stress, we have discovered several promising commercially available multi-sensor systems. Smart health watches are very widespread in the today’s population. Since early 2010s, the CPU computational power and the overall performance have been high enough to run sophisticated machine learning algorithms, and such devices are able to derive stress from HR. Actually, the most popular way to detect stress today is using wrist wearables. WHOOP’s Recovery metric uses HRV, resting HR, sleep and respiratory rate to determine the state of recovery after body underwent stressful endurance physical training [189]. Nowadays, smartwatches and trackers from Garmin [190] such as Vivoactive 4, Vivosmart 4, Fenix 6, from Samsung [191] Galaxy Watch and Galaxy Watch Active 2, from Apple Watch Series 6 [192], Google Wear smartwatches [193] such as Fossil Gen 5, Fossil Sport, TicWatch E2 and Skagen Falster 3 and Fitbit products [194,195] such as Charge 4, Versa 3 and Ionic are worth mentioning devices on the market. An interesting solution from the Fitbit Company in the Sense product is the possibility to measure also the SCL of EDA after placing your second hand over the watch thanks multi-path electrical sensor [194]. Using Fitbit Sense, you can make a mental well-being practice. Clinical studies have shown that such meditation is very effective in reducing stress [33]. Last but not least, Withings Scanwatch [196] with medical grade ECG and oximeter and two devices from company Empatica: Embrace 2 and E4 should be mentioned [197,198,199]. Both are in the form of wristband and can stream the following variables in real time: EDA, wrist temperature and accelerometric signal. E4 is enhanced further by employing a PPG sensor. Embrace 2 was especially designed for epilepsy monitoring, sleep/rest management and physical activity tracking. E4 is more suitable for lab or home recording, real-time clinical observation and raw data analysis. Very interesting is also Samsung health device concept Simband with Simsense [200], which includes PPG, EDA, skin temperature, 3D accelerometer and ECG lead. Today’s advances in miniaturization allow us to monitor physiology with a ring. Perfect example is the Oura health ring, which includes PPG and temperature sensors together with an accelerometer and a gyroscope encapsulated in an attractive package [201]. Another very common form of recording human physiology are chest-belts. A typical representative is, for example, Zephyr Bioharness 3 that includes HR, RR and accelerometric monitoring [202]. One can also appreciate the design of the non-traditional Qardiocore chest holter [203] that captures ECG, RR, temperature and activity recognition, as well as Wearable biosensor from Philips [204], which contains an ECG sensor together with an accelerometer and a skin temperature sensor. A chapter in themselves are headband neurotrackers like Neurosky Mindwave [205], Muse 2 EEG [206], and Flowtime [207] that monitors EEG and helps to reduce stress through meditation. Muse 2 and Flowtime have also an integrated PPG sensor. Spire Health Tag [208] is also worth mentioning. This device can be adhered inside clothes and detect heart rate, breathing patterns, and body movements to assess the emotional and mental well-being of a person. Detailed parameters of the selected wearable devices suitable for stress-detection can be found in Table 1. Within some experiments, scientists have developed their own designs. One of these offers stress detection from portable ECG and EDA, where authors use two channels EDA and ECG to suppress undesired artifacts [108]. Another example is the activity recognition system (mental, physical and emotions) based on combination of ECG and respiration sensor, and EDA gloves [209]. Systems using speech analysis also appear to be very promising for stress analysis purposes. These systems can be easily incorporated into modern mobile phones [210]. In the research of Jin et al. [42], in addition to the speech analysis itself, they included behavioral signals (3D accelerometer and 3D angle sensor) and propose an attention-based deep-learning architecture for a more accurate classification of mental state. Nowadays, there are even systems that perform the analysis of biomarkers from sweat and saliva directly during sports activities [211]. The current literature summary of stress assessment using wearable multi-sensors in the natural environment includes: emotion recognition by neural networks from portable eyetracker and Empatica E4 [212], ANS research using again E4 but now with ECG and respiration sensors [213], development of cognitive load tracker using machine learning [109], smart stress reduction system using E4 combined with accelerometers [214], validation of wireless sensors for psychophysiological studies and stress detection [100,215], prediction of relative physical activity [216], real-time monitoring of passenger psychological stress [147], classification of calm/distress condition [217], assessment of mental stress of fighters [218], and others. A comprehensive overview about pain and stress detection using available wearable sensors was actually made very recently by Jerry Chen et al. [150]. They mention, stress monitoring using mobile EEG head set MindWave [219], ECG and EMG DataLOG [220], using a combination of MindWave EEG (NeuroSky, San Jose, CA, USA), Zephyr BioHarness 3 chest belt (Medtronic, Boulder, CO, USA), Shimmer Sensor (Shimmer Sensing, Dublin, Ireland) [221] and mobile sensors suite AutoSense (National Institutes of Health, Bethesda, MD, USA) [222]. Mental health monitoring using ubiquitous wearable sensors [223] and machine learning [224,225] has been also described.

### 4.2. The Proposed Multi-Sensor Concept

**Figure 7:**
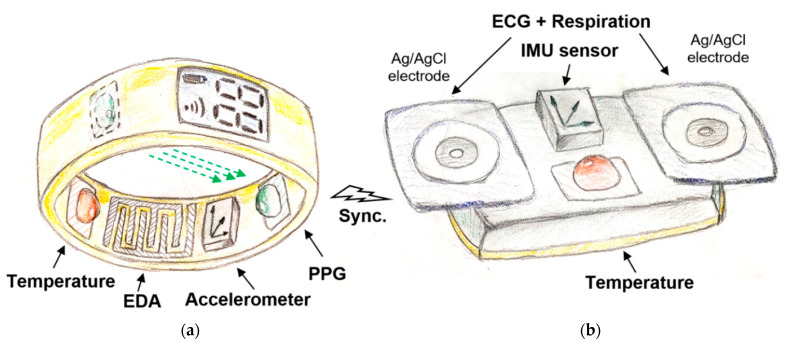
Concept of new stress-meter: (**a**) smart ring, (**b**) chest holter.

Based on the analysis described in the previous sections, we still see points where current multi-sensor monitoring systems can be improved, and therefore we come up with our own concept. From practical reasons, the main idea behind the concept is to divide the stress-meter system into two separate sub-systems (Figure 7). We consider appropriate to place one sub-system on bottom parts of non-dominant hand, ideally on fingers (smart-ring) as depicted in Figure 7a. The second part of sensors should be placed on the chest in the heart area, most probably in the form of a smart sticker or a tiny chest belt, as depicted in Figure 7b. The keystone lays in the precise time synchronization of those two sub-systems.

Let us start with a smart ring illustrated in Figure 7a. The basis component, as in most other stress detection devices, should be an exosomatic EDA sensor stored in a smart-ring (Figure 7a). For practical reasons of long-term monitoring, we choose dry electrodes and harmonic signal. However, instead of conventional macroelectrodes, we recommend using optimized microelectrodes in the form of symmetrical IDA microelectrodes of dimensions between 100/100 and 200/200 μm (finger/gap). In this way, we will be able to obtain the EDA signal from a smaller area and the measure signal will be more tied to nerve activity and stress. In the first research phases, we would not even ignore the possibility of measuring the complex impedance at different frequencies. From our experience a harmonic input signal with the frequency value about 1 kHz and the amplitude between 1.5 and 3 V is recommended. Since dry microelectrodes are intended to be used, there will be a need to minimize the motion artifacts. Despite the fact that we have high hopes for software filters, built in circuity as a part of a standard physical sensor is assumed. A simple accelerometer seems to be the most suitable choice, but using the piezoelectric pressure sensor we can obtain direct information about the contact force between the electrodes and the skin. Another essential part of this sensor sub-system must be the optical HR sensor, where conventional PPG sensor can be used. As confirmed in the previous analysis, monitoring of blood oxygenation is not necessary, but at the present COVID-19 time, will certainly not be redundant. The transmittance principle of PPG used on fingers has been suggested in [226], which is more reliable compared to the reflective principle. The last sensor in the ring/watch sub-system should be a reliable thermometer, whether it is a contact thermocouple or an optical one. It should not be a problem to place all these sensors in a still compact ring of about 1.5 cm thickness. An area of about 2 cm^2^ is sufficient for EDA electrodes, LED and photodiode placed opposite each other and the thermal sensor occupy only a few tens of mm^2^. The battery and the transmitter are the largest parts. If there is a requirement for further integration, we have experience with the use of transparent metals for EDA electrodes [116]. Such a design would allow the placement of optical sensors directly behind EDA electrodes.

The second multi-sensor sub-system (Figure 7b) should definitely contain an ECG sensor. The quality of the ECG itself is not important, we are dominantly interested only in the exact estimate of the R peak in the QRS complex. From this information we obtain HR and, thanks to the precise synchronization with the first sensor sub-system and the PPG, also pulse transition time (PTT) which is relative to BP. If the quality of ECG is high enough, it will definitely not hurt. ECG holters are the most commonly used telemedicine devices and they can also monitor other cardiovascular parameters that can more accurately determine the physiological condition. We have good experience with chips from Texas Instruments (TI 1292R) [227], which also integrate a reliable respiration impedance sensor, so in this way, the second important physiological parameter—the respiration could be obtained. Biosignals can be measured using Ag/AgCl adhesive electrodes with the sufficient mutual distance of 5 cm. Another important sensor is an IMU sensor (3-axis accelerometer, gyroscope and compass). There are an infinite series of IMU sensors. Since the IMU sensor will be placed on the chest, the best overview of posture and physical activity of the body can be extracted. An IMU sensor on your hands or feet would not be reliable enough. Information on overall human activity will help us better understand the very important phasic response of the EDA signal. We know that in some situations, especially with higher physical activity, the signal from the accelerometer so-called seismocardiographic signal, can replace the ECG signal in determining HR [141]. The last used sensor here is again a body core thermal sensor.

From the all sensors used, the following physiological variables will be obtained: ECG and chest temperature, HR and finger temperature, EDA, EDA motion artefacts, respiration, posture and physical activity. Synchronization is now entering the scene. For sub-systems synchronization and external communication we plan to use Bluetooth Low Energy. By comparing the temperature of the finger and chest, one can get information about the heat gradient and so-called “circulating shock” level. From the determination of the time shift between the ECG and PPG signals, continuous information about the blood flow rate will be available, which in our case can be considered as BP. Here is the key to accurate synchronization. We estimate that an inaccuracy of 5 ms corresponds to a blood pressure error of 1 mmHg corresponding to the average person when using a ring. We should try to achieve a sync error of about 1 ms [228]. Additional information on systolic and diastolic pressure can also be obtained by analyzing the shape of the PPG curve [124,141,229]. In addition to bluetooth connectivity, the concept can be also connected via USB. The device will have integrated an internal memory (micro SD card), real-time clock and battery management circuitry. The battery management is one of issues to be investigated further. Here, energy harvesting systems generating electrical energy from ambient environment represent a promising solution towards enhancing the battery lifetime or avoiding the necessity of battery change that might be very impractical and inconvenient in some applications [230,231] could be effectively employed to provide a self-powered (at least partially) electronic system. Detailed parameters of proposed multi-sensor concept are summarized at the end of Table 1.

After designing a device, which is the main development goal in the near future, the experimental part will follow. The biggest challenge will be to examine the coherence of all the measured physiological variables during different situations in human life. Some mutual relations have been already analyzed within the previous analysis, however, skillful programmers and the use of neural networks and machine learning will be in high demand. Of course, one cannot avoid laboratory and simulated situations supplemented by standard psychological tests and stress assessments, whether by standardized questionnaires or by measuring hormone levels to fine-tune the device.

## 5. Conclusions

We live in a world where there are a lot of stressors around us. Everyone is stressed from something else, but in the end, the body’s reactions and its physiological manifestations are rather the same. These common manifestations can be measured and their recording determines the body’s response to stress. When a person is better aware of living under stress, they can recover by physical movement, relaxation or otherwise relieve the stress. Our goal was to find out what are the latest approaches and systems for measuring human physiology, and to design a multi-sensor device for measuring acute and chronic stress. For this purpose, we performed a detailed review of measurements of selected physiological variables, which we consider appropriate to use in a precise wearable stress meter and also presented several promising existing wearable devices. Compared to simple devices, which mostly determine stress based on one or a few variables, we realize that a reliable device for daily stress monitoring has to consist of a coherent set of sensors in order to form a wearable lie detector device. The proposed concept will uniquely use multiple physiological variables and measure these variables using two synchronized sensor blocks to improve the monitoring quality and interpretation of results. The main benefits of the proposed concept for average commercial facilities include: the use of IDA microelectrodes with higher sensitivity for EDA measurement, possibility to examine complex impedance of EDA, and hardware tracking of motion artifacts. Using the chest sub-system we will get continuous high quality single channel ECG, respiration derived from chest impedance, and adequately monitoring of posture and physical activity. In the ring sub-system, we can apply a more reliable PPG transmittance sensor. Thanks to the synchronization of the continuous BP signal of both sub-systems and using two temperature sensors, we can monitor the peripheral skin temperature and the body core temperature at the same time and to determine the body’s heat gradient. In the concept, we also consider the use of energy harvesting.

We do believe that this proposal will attract researchers and designers in this field. In any case, we will develop, implement, verify and optimize the proposed concept in the near future. Part of our research is also the implementation of the presented techniques into smart clothing.

## Figures and Tables

**Table 1:** Technical parameters of selected wearable devices suitable for stress-detection.

**Garmin Vivoactive 4** [190]
*Sensors*: PPG ^1^, accelerometer, gyroscope, compass, barometer, thermometer, microphone, ambient light, GPS, GLONASS, Galileo
*Physiology variables*: HR, spO_2_, stress ^2^, respiration ^2^ (meditation), sleep and activity tracking, body battery energy
*Connectivity*: Bluetooth, Wi-Fi, NFC, ANT+, *Waterproof*: 5 ATM; *Form*: Watch
**Samsung Galaxy Watch Active 2** [191]
*Sensors*: PPG ^1^, ECG ^3^, accelerometer, gyroscope, compass, barometer, microphone, ambient light, GPS, GLONASS, Galileo, Beidou
*Physiology variables*: ECG ^3^, HR, BP ^3^, stress ^2^ (meditation), sleep and activity monitoring
*Connectivity*: Bluetooth, Wi-Fi, NFC; *Waterproof*: 5 ATM; *Form*: watch
**Google Fossil Gen 5** [193]
*Sensors*: PPG ^1^, accelerometer, gyroscope, compass, barometer, microphone, ambient light, GPS
*Physiology variables*: HR, stress ^2^ (meditation), sleep and activity monitoring
*Connectivity*: Bluetooth, Wi-Fi, NFC; *Waterproof*: 3 ATM; *Form*: watch
**Apple Watch 6** [192]
*Sensors*: PPG ^1^, ECG ^3^, accelerometer, gyroscope, compass, barometer, microphone, ambient light, GPS, GLONASS, Galileo
*Physiology variables*: ECG ^3^, HR, spO_2_, sleep and activity monitoring
*Connectivity*: Bluetooth, Wi-Fi, NFC; *Waterproof*: 5 ATM; *Form*: watch
**Withings Scanwatch** [196]
*Sensors*: PPG ^1^, ECG ^3^, accelerometer, gyroscope, compass, barometer, ambient light
*Physiology variables*: ECG ^3^, HR, spO_2_ (medical grade), sleep and activity tracking
*Connectivity*: Bluetooth Low Energy, USB; *Waterproof*: 5 ATM; *Form*: watch
**Fitbit Sense** [195]
*Sensors*: PPG ^1^, ECG ^3^, EDA ^3^, thermometer, accelerometer, gyroscope, barometer, microphone, ambient light, GPS, GLONASS
*Physiology variables*: ECG ^3^, HR, spO_2_, peripheral temperature, stress (meditation), sleep and activity monitoring
*Connectivity*: Bluetooth Low Energy, Wi-Fi, NFC; *Waterproof*: 5 ATM; *Form*: watch
**Samsung Simband** [200]
*Sensors*: PPG ^1^, ECG ^3^, EDA, bioimpedance, thermometer, accelerometer
*Physiology variables*: ECG ^3^, HR, EDA, bioimpedance (blood flow), peripheral temperature, activity tracking
*Connectivity*: Bluetooth, USB; *Form*: watch
**Empatica Embrace 2** [199]
*Sensors*: EDA, temperature, accelerometer, gyroscope
*Physiology variables*: EDA (clinical grade), peripheral temperature, stress, sleep and activity tracking
*Connectivity*: Bluetooth Low Energy; *Waterproof*: 0.1 ATM; *Form*: wristband
**Empatica E4** [199]
*Sensors*: PPG ^1^, EDA, infrared temperature, accelerometer, event maker
*Physiology variables*: HR, spO_2_, EDA, peripheral temperature, activity tracking
*Connectivity*: Bluetooth Low Energy, USB 2.0, Raw data analysis; *Waterproof*: 0.1 ATM; *Form*: wristband
**Zephyr Bioharness 3** [202]
*Sensors*: HR, RR, accelerometer
*Physiology variables*: HR, RR, activity monitoring
*Connectivity*: Bluetooth Low Energy; *Form*: chest-belt
**Quardiocore** [203]
*Sensors*: ECG, skin temperature, accelerometer, gyroscope, compass
*Physiology variables*: ECG, HR, RR, body core temperature, activity tracking
*Connectivity*: Bluetooth 4.0; *Form*: chest-belt
**Philips Wearable biosensor** [204]
*Sensors*: ECG, skin temperature, accelerometer
*Physiology variables*: ECG, HR, RR, body core temperature, activity tracking
*Connectivity*: Bluetooth; *Form*: chest-belt
**Oura health ring** [232]
*Sensors*: PPG ^4^, accelerometer, gyroscope, NTC thermometer
*Physiology variables*: HR, RR ^2^, peripheral temperature, stress (meditation), sleep and activity tracking
*Connectivity*: Bluetooth Low Energy; *Waterproof*: 10 ATM; *Form*: ring
**Neurosky MindWave** [205]
*Sensors*: EEG
*Physiology variables*: EEG
*Connectivity*: Bluetooth/Bluetooth Low Energy dual mode; *Form*: headband
**Muse 2 EEG** [206]
*Sensors*: EEG, PPG ^1^, accelerometer, gyroscope
*Physiology variables*: EEG (emotions), HR, stress (meditation) and activity tracking
*Connectivity*: Bluetooth 4.2, USB; *Form*: headband
**Flowtime EEG** [207]
*Sensors*: EEG, PPG ^1^
*Physiology variables*: EEG, HR, stress tracking (active/neutral/calm)
*Connectivity*: Bluetooth; *Form*: headband
**Spire Health Tag** [208]
*Sensors*: PPG ^1^, accelerometer
*Physiology variables*: HR, RR ^2^, stress (calm/focus/tension), sleep and activity tracking
*Connectivity*: Bluetooth; *Form*: adhered to clothes; *Washer and dryer proof*
**Multi-sensor concept**
*Sensors*: ECG, PPG ^4^, respiration, EDA (IDA microelectrodes), 2× infrared temperature, 2× accelerometer, gyroscope, compass
*Physiology variables*: ECG, HR, spO_2_, EDA, respiration, peripheral and body core temperature (heat gradient), BP (derived from ECG and PPG), stress, sleep and activity monitoring
*Connectivity*: Bluetooth Low Energy, USB; *Form*: ring and chest-belt

^1^ Reflectance principle. ^2^ Derived from HRV. ^3^ On demand—second hand must be placed on device. ^4^ Transmittance principle.

## Data Availability

The data presented in this study are available on request from the corresponding author.
